# Gamma probe-assisted excision of an ectopic parathyroid adenoma located within the thymus: case report and review of the literature

**DOI:** 10.1186/1749-8090-9-62

**Published:** 2014-03-31

**Authors:** Stavros I Daliakopoulos, George Chatzoulis, Savvas Lampridis, Varvara Pantelidou, Omiros Zografos, Konstantinos Ioannidis, Michael Sapranidis, Avraam Ploumis

**Affiliations:** 1 Department of Thoracic Surgery, 424 General Military Hospital, Eukarpia Ring Road, Thessaloniki Gr 564 29, Greece; 2 Department of Surgery, 424 General Military Hospital, Eukarpia Ring Road, Thessaloniki Gr 564 29, Greece; 3 Department of Endocrinology, Hippokration Hospital, Konstantinoupoleos 49, Thessaloniki Gr 546 42, Greece; 4 Department of Orthopaedic Surgery and Rehabilitation, University Hospital of Ioannina, Stavrou Niarchou Avenue, Ioannina Gr 45 500, Greece

**Keywords:** Ectopic parathyroid adenoma, ^99m^Tc sestamibi, Gamma probe

## Abstract

Primary hyperparathyroidism due to parathyroid adenomas may be associated with ectopic parathyroid gland localization in 20-25% of the patients. We report herein the excision of an ectopic parathyroid adenoma which was detected in the thymus gland by gamma probe intraoperatively. A 38-year-old patient presented to our clinic with a history of bilateral nephrolithiasis, chronic hypercalcaemia, and PTH elevation. A combination of Technetium-99 m sestamibi scintigraphy and Computed Tomography scan of the chest and neck revealed an ectopic parathyroid adenoma of 8.5 mm in its greatest dimension. The patient underwent sternotomy and the adenoma was found within the right lobe of the thymus gland with the intraoperative use of gamma probe. PTH detection and frozen biopsy were performed during surgery and confirmed the successful excision of the adenoma, while mild hypocalcaemia was noticed postoperatively. We conclude that accurate preoperative and intraoperative localization of an ectopic parathyroid adenoma is crucial to successful surgery. The use of at least two diagnostic modalities before surgical excision minimizes the risk of re-operation for recurrent hyperparathyroidism, while the intraoperative use of gamma probe offers a significant advantage over conventional techniques by reducing surgical time, morbidity and/or complications associated with surgical exploration.

## Background

Primary hyperparathyroidism is caused by parathyroid adenomas up to 80% of the cases. 20% of these present as ectopic parathyroid glands during embryogenesis and the majority of them are located near or within the thymus due to the common origin of the thymus and the inferior parathyroid glands from the third bronchial pouch [1]. Possible positions of the embryologically ectopic adenoma is the deep anterior mediastinum, the aortopulmonary window, the posterior mediastinum, or within the substance of the thyroid gland [2]. However, paraesophageal and retroesophageal parathyroid tumors are not considered ectopic since they have normal blood supply from a branch of the inferior thyroid artery and they arise from superior parathyroid glands [3].

Preoperative diagnostic methods for the localization of primary parathyroid adenomas have their advocates and opponents. Many studies have confirmed the efficacy of Technetium-99 m (^99m^Tc) sestamibi scans in identifying mediastinal adenomas [2]. It was first designed and in the last 20 years clinically implemented by Coackley et al. [4]. This method localizes the tumor in 85-90% of cases and simplifies the surgical management. In case of ectopic location of a parathyroid adenoma, in 1996 Sofferman et al. reported exact preoperative localization with scintigraphy at a high rate of 90% [5]. Even though the routine use of scintigraphy is cost effective in non-ectopic parathyroid adenomas, in the case of an ectopic adenoma it is precious, since it cannot be detected by conventional cervical exploration. Preoperative localization should be defined with Computed Tomography (CT), Magnetic Resonance Imaging (MRI) or Single Photon Emission Computed Tomography (SPECT). Moreover, the gamma probe–guided parathyroidectomy is a very accurate surgical approach to treat patients with primary hyperparathyroidism due to an ectopic solitary parathyroid adenoma. Additionally, the intraoperative frozen biopsy with PTH assays that accounts for a 50% or more decline from baseline within 10 minutes of excision confirms the successful surgical exploration and excision [6].

We present the case of an ectopic parathyroid adenoma located inside the thymus gland which was successfully managed surgically, with the concomitant assistance of gamma probe.

## Case presentation

A 38-year-old Caucasian male was admitted to the Department of Surgery because of an ectopic parathyroid adenoma. He also had a history of chronic hypercalcaemia and symptomatic nephrolithiasis which were closely followed by the Department of Endocrinology for control and regulation of serum parathyroid hormone and calcium.

**Figure 1:**
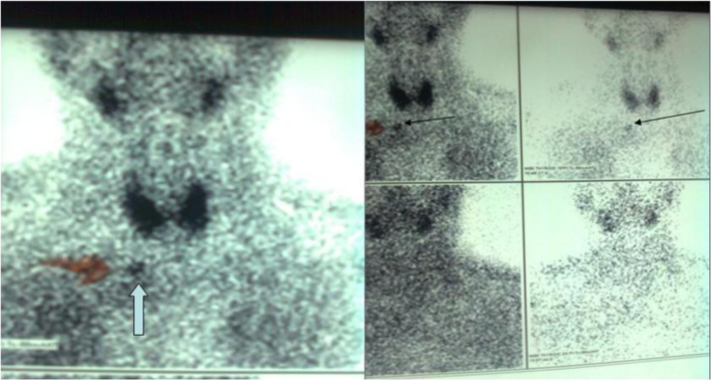
**Thyroid-parathyroid sestamibi scan image of an ectopic parathyroid adenoma.** A thyroid-parathyroid sestamibi scan image showing an ectopic parathyroid adenoma below the lower pole of the thyroid gland, in a possible intrathoracic position.

**Figure 2:**
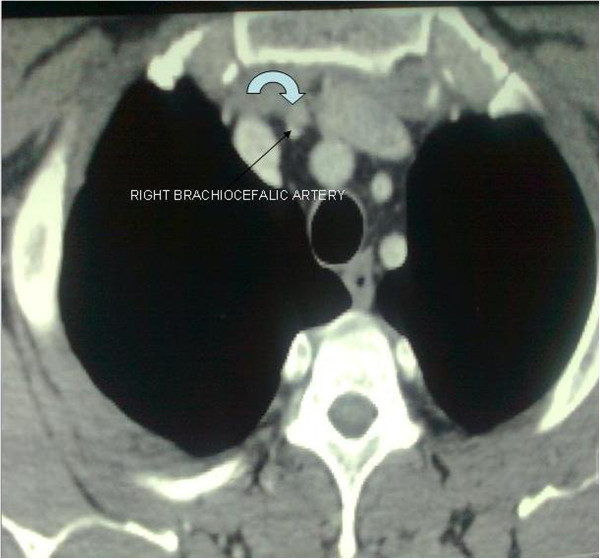
**Chest CT of a cervical intrathoracic nodule.** Chest CT revealing a cervical intrathoracic nodule, about 1 cm in diameter, in close contact with the thymus gland.

Four months before surgery an abdomen CT and a pyelogram showed a urolith of 1.5 cm maximum diameter in the left renal pelvis and 2–3 smaller uroliths in the lower pole of the left kidney with mild hydronephrosis. At the same time, a thyroid-parathyroid sestamibi scan revealed an ectopic parathyroid adenoma below the lower pole of the thyroid, probably inside the thorax (Figure 1). In a thyroid ultrasound, a nodule 8.7 × 5.7 cm with internal vascularization was seen. Three months preoperatively the patient underwent an extracorporeal lithotripsy. At that time, a chest CT revealed a cervical intrathoracic nodule close to the thymus gland, about 1 cm in diameter, and multiple lymph nodes in the posterior cervical triangle bilaterally, while no swollen lymph nodes were recorded in the mediastinum (Figure 2). Laboratory findings one month before surgery were: serum calcium (Ca): 12.1 mg/dl (normal range: 8.5-10.2), 24-hour urine Ca: 316 mg/dl (normal range: 25–300), serum sodium (Na): 141 mmol/l (normal range: 135–145), serum urea: 25 mg/dl (normal range: 20–45), serum creatinine: 0.86 mg/dl (normal range: 0.72-1.25), parathormone (PTH): 164 pg/ml (normal range: 8–51). Preoperative laboratory findings were: hematocrit: 47.6% (normal range: 41–50), white cell count: 9.3 × 10^3^/μl (normal range: 4.2-11 × 10^3^), serum Ca: 12.3 mg/dl, phosphorus (P): 2.1 mg/dl (normal range: 2.3-4.7), alkaline phosphatase (ALP): 130 U/L (normal range: 40–150), serum urea: 30 mg/dl, serum creatinine: 0.78 mg/dl, triiodothyronine (T_3_) total: 0.6 ng/ml (normal range: 0.6-2), thyroxine (T_4_) total: 3.03 μg/dl (normal range: 3.2-12), thyroid stimulating hormone (TSH): 1.08 μUI/ml (normal range: 0.27-4.70). PTH just before surgery was 171.6 pg/ml (baseline). Preoperatively, a ^99m^Tc sestamibi scan with 740 mBq (20 mCi) was performed and planar imaging of the neck and chest was taken on a dual head gamma camera after a delay of 20 minutes and repeated after 2 hours. This test demonstrated a mild uptake of the sestamibi in the neck region with concomitant increased uptake in the upper anterior mediastinum in the delayed phase.

**Figure 3:**
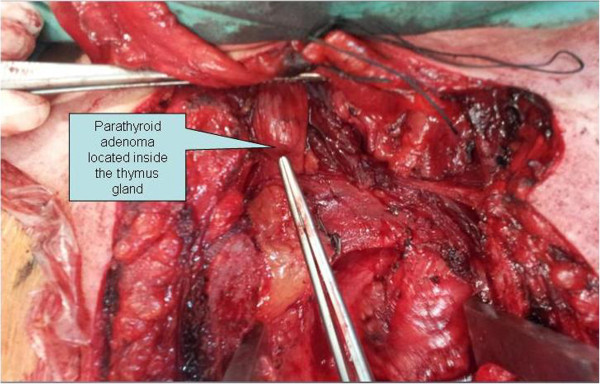
**Intraoperative image of an ectopic parathyroid adenoma inside the thymus gland.** An intraoperative image, after a median sternotomy, of an ectopic parathyroid adenoma located within the thymus gland.

During the operation, the patient initially underwent bilateral exploration of the lower pole of the thyroid in the neck and then, at the same stage, a median sternotomy (Figure 3). The use of gamma-probe radioactivity consisted of monitoring the thyroid background as a guide, whereas within the thymus high pitch signals in a ratio parathyroid/thyroid = 2.5 were recorded. The gamma probe is a hand held probe containing a radiation detector, providing a count rate from gamma rays. This hand held probe is connected to a power supply and a unit that receives the electrical audible high pitch signals that come from the radiation detector. Subsequently, the parathyroid adenoma was removed as it was located inside the thymus gland. The intraoperative documentation of the existence of adenoma was made by three ways: a) The intraoperative use of gamma probe with increasing signals of the gamma-probe output (the high-pitch signals produced by the gamma probe) when the probe was in contact with parathyroid adenoma in vivo or ex vivo producing high radioactivity. b) The frozen biopsy of the parathyroid adenoma. c) The intraoperative PTH serum value (after removal of ectopic parathyroid adenoma) was 31 pg/ml (80% below from baseline). Operative time was approximately 1.5 hours.

Immediately postoperatively, serum Ca subsided to 10.1 mg/dl. The postoperative course was uneventful. There was also a significant but transient postoperative decline of serum Ca to 8 mg/dl with no confirmative evidence of the “hungry bone” syndrome (phosphorus: 4.5 mg/dl). The patient was discharged on the 13th postoperative day (at discharge, serum Ca: 8.9 mg/dl and ALP: 89 U/L).

## Conclusion

The definition of primary hyperparathyroidism is the hypersecretion of parathyroid hormone by one or more parathyroid adenomas [7]. The incidence of primary hyperparathyroidism in the United States is about 0.5 per 1,000 (51% asymptomatic) with a predominance of women > 40 years, and in Europe 3 per 1,000 total, reaching to 21 per 1,000 in women 55–75 years old [8].

The 95% of the body calcium presents in the form of calcium phosphate or hydroxyapatite [Ca5(PO4)3OH], with main regulatory depots being the teeth and bones where the ionized calcium is maintained in a ratio of 1 mmol/l (or 4 mg/dl). On the other hand, parathyroid hormone (PTH) is a polypeptide regulator of calcium ion concentration in blood and extracellular fluid, where it is metabolized by the liver (70%) and kidneys (20%) and where 1% participates in reactions with cell-surface PTH receptors rooted in bone and kidney, causing an increase in blood calcium. Thus, PTH hypersecretion, caused by a parathyroid adenoma, hyperplasia, or carcinoma, leads to increase of the extracellular calcium [7].

The clinical manifestations (hypercalcemia and bilateral nephrolithiasis) of our patient were typical for hyperparathyroidism. Generally the symptoms of primary hyperparathyroidism are characterized by recurrent nephrolithiasis and hypercalcemia with low level of blood phosphorus, bony defect, weakness, and low life expectancy. However mild hyperalcemia is combined with less severe symptoms [7].

The preoperative diagnostic tools to detect parathyroid adenomas consist of ultrasound (US), CT, and MRI with accuracy 57-68% depending on the size and location of these adenomas [9]. The preoperative diagnosis of adenomas by ultrasound presents with a sensitivity range between 70% and 80% [10,11], while this ratio regrets to 40% in patients who underwent previous failed surgical exploration [12]. Localizing parathyroid adenomas with ultrasound has high success rates when adenomas are located behind the thyroid lobes, while it is difficult to locate them when they are near to the larynx and trachea, in the bifurcation of the carotid artery and in the para- and retropharyngeal space [7]. Moreover the combination of US-guided fine-needle aspiration and thyroid scintigraphy in the same session is valuable in the detection of parathyroid adenomas within thyroid nodules by recording the increase in the size of the parathyroid glands especially in the case of secondary hyperparathyroidism [13,14]. CT and MRI have significant value in the ectopic location of parathyroid adenomas. An excellent overall sensitivity of about 78% is provided by a three-dimensional MRI of the neck and upper chest. The sensitivity is higher (88%) in the mediastinum. This percentage is equivalent to that of dual-phase ^99m^Tc sestamibi scintigraphy [15]. More recently simultaneous PET (Positron Emission Tomography) and MRI is a new hybrid method of imaging that provides excellent soft-tissue contrast and high imaging quality of ectopic mediastinal parathyroid adenomas [16]. More specifically, CT is used for the localization of parathyroid adenomas located in the retrotracheal, retroesophageal, and mediastinal spaces with higher resolution than the US and presents difficulty in diagnosis when adenomas are found near or within the thyroid gland, with an overall sensitivity range between 46% and 80% [17]. In our case, a CT thorax established the location of an ectopic parathyroid adenoma inside the thymus gland.

Coakley et al. reported a random observation of increased absorbance of ^99m^Tc sestamibi from dysfunctional parathyroid glands during a study of vascular perfusion of the myocardium [4]. Three entities correlate with increased uptake of ^99m^Tc sestamibi in parathyroid glands: a) the size of the parathyroid glands, b) the amount of blood flow and c) the mitochondrial energy potential [18]. It is noticeable that ^99m^Tc sestamibi is cleared first by the thyroid gland and subsequently by the parathyroid glands making it specific for imaging of parathyroid adenomas. This difference in the time of ^99m^Tc sestamibi wash out has a pathophysiologic basis in the dysfunction of P-glycoprotein system [19]. Thus, the scintigraphy with ^99m^Tc sestamibi can detect parathyroid adenomas with a diagnostic accuracy of 85%-95%. The combination of CT and ^99m^Tc sestamibi has 100% sensitivity and 97.4% positive predictive value for the detection of ectopic parathyroid adenomas [20]. Regarding localisation of ectopic parathyroid adenomas, such as in the posterior mediastinum, valuable help is provided by using SPECT and especially the dual phase SPECT/CT [21,22]. Fluoride DeoxyGlucose-Positron Emission Tomography (FDG-PET) is a cost effective diagnostic modality of ectopic parathyroid adenomas associated with a higher sensitivity [23].

The use of gamma probe intraoperatively and 2–3 hours after the injection of ^99m^Tc sestamibi, is accurate for the localization of ectopic adenomas. The location with the highest radioactivity is characterized by audible high pitch signals produced by gamma probe. By using the thyroid tissue radioactivity as background, a parathyroid-to-thyroid ratio higher than 1.5 strongly suggests the presence of a parathyroid adenoma. Standard bibliographic reports for parathyroid-to-background ratios (excluding the thyroid tissue) range between 2.5 and 4.5. In our case, there was a 2.5 ratio between thymus location and thyroid background, being the same intraoperatively as well as in ex vivo position. It is remarkable that the ex vivo counting rate of an adenoma is at least 20% and usually is 50% higher than the thyroid background (20% rule) [24]. In our case, the percentage was much higher than 50%. After the adenoma excision the surgical bed is scanned for a new level of background radioactivity. Another criterion for a successful excision of parathyroid adenoma is a ratio greater than 1.2 between the ex vivo lesion counts and the residual background counts which was strongly consistent with our case as the aforementioned ratio was 1.8 [7].

Bilateral neck exploration during parathyroidectomy includes the dissection of the inferior thyroid artery’s bifurcation, the posterior surface of the thyroid lobe, the retropharyngeal and thyro-carotid spaces, the thymic and upper mediastinal area, and the posterior segment of the trachea and esophagus [1]. Gilmour et al. recorded an anatomical abnormality related to the presence of more than four glands and combining appearance of ectopic parathyroid gland inside the mediastinum. Ectopic parathyroid adenomas are found in the mediastinum within the thymus gland, like in this case, at a ratio of 1-2% [25,26]. Median sternotomy and mediastinoscopy (video assisted thoracoscopic surgery or VATS) with the intraoperative use of gamma probe are both effective for parathyroid adenoma excision [27]. Quick parathyroid hormone (QPTH) is a very helpful test in parathyroid surgery as the recording of a 50% drop from the preoperative parathyroid baseline level 10 min after excision, is a marker of successful adenoma excision (the Miami criterion) [28]. QPTH is also used for exclusion of multiglandular parathyroid disease 1%-3.5% at the same intraoperative session [29]. An alternative therapeutic approach, in patients who cannot be operated, is the angioablation with injection of a hyperosmolar agent [30]. Since there are reports of failed surgery in ectopic adenomas without the use of gamma probe the latter becomes the mainstay for exact adenoma detection [2]. Usually the cause of a failed primary exploration is an occult double adenoma which necessitates the combination of gamma probe and QPTH in the intraoperative detection of the ectopic parathyroid adenoma. Nevertheless, a gamma probe assisted surgical exploration, as the only method, has a reported high success rate of 98% [31]. The full surgical time was 90 minutes, while two procedures were performed: a bilateral lower thyroid pole exploration in order to exclude multiglandular parathyroid disease (40 minutes) and a median sternotomy (50 minutes). Considering the bibliographic references of a prolonged surgical time without the use of radio-guided localization (median time 90–112 minutes), gamma probe is precious for parathyroid adenomas [32-35]. Another report emphasizes that the number of patients with persistent hyperparathyroidism after surgery increased significantly from 0.8% to 5.0% without measuring intra-operative QPTH [36]. Since a failed surgery involves large cost effects, a meticulous preoperative imaging with ultrasound, ^99m^Tc sestamibi, and MRI or CT is mandatory for the primary and moreover the revision surgery [2].

No signs for persistent hypocalcemia and “hungry bone” syndrome were noticed in this case. Regarding the latter the postoperative serum Ca level fell temporarily at 8.1 mg/dl with normal values of phosphorus. Serum Ca values lower than 8.5 mg/dl and phosphorus of less than 3 mg/dl on the third postoperative day is typical of “hungry bone” syndrome. This syndrome is characterized by persistent hypocalcaemia, hypophosphatemia and hypomagnesaemia due to the fall in PTH levels and occurs in primary and secondary hyperparathyroidism, with the second most frequently. It is remarkable that higher values of preoperative PTH, Ca, and ALP are predictors of higher risk for this syndrome [37].

Conclusively, preoperative localisation of an ectopic parathyroid adenoma with the use of CT and ^99m^Tc sestamibi is accurate and gamma probe-assisted excision leads to complete removal of the adenoma verified by intraoperative use of QPTH and frozen biopsy.

## Consent

Written informed consent was obtained from the patient for publication of this case report and any accompanying images. A copy of the written consent is available for review by the Editor-in-Chief of this journal.

## Abbreviations

99mTc: Technetium-99 m; ALP: Alkaline phosphatase; Ca: Calcium; cm: Centimeter; CT: Computed tomography; dl: Deciliter; FDG-PET: Fluoride DeoxyGlucose-Positron emission tomography; l: Liter; mBq: Millibecquerel; mCi: Millicurie; mg: Milligram; mmol: Millimole; MRI: Magnetic resonance imaging; Na: Sodium; P: Phosphorus; PET: Positron emission tomography; PTH: Parathormone; QPTH: Quick parathyroid hormone; SPECT: Single photon emission computed tomography; T3: Triiodothyronine; T4: Thyroxine; TSH: Thyroid stimulating hormone; US: Ultrasound; VATS: Video assisted thoracoscopic surgery; μl: Microliter.

## Competing interests

The authors declare that they have no competing interests.

## Authors’ contributions

SID, GC, SL, and VP performed surgery and prepared the manuscript. OZ, KI, and MS participated in the design of the case report and aided in literature search. AP coordinated the preparation of the case report. All authors read and approved the final manuscript.
